# Computer-aided design of polyetheretherketone for application to removable pediatric space maintainers

**DOI:** 10.1186/s12903-020-01184-6

**Published:** 2020-07-10

**Authors:** Hui Guo, Yong Wang, Yijiao Zhao, He Liu

**Affiliations:** 1 Department of Pediatric Dentistry, Peking University School of Stomatology, 22 Zhongguancun Nandajie, Haidian District, Beijing, 100081 China; 2 National Engineering Laboratory for Stomatology Digital Technology, 22 Zhongguancun Nandajie, Haidian District, Beijing, 100081 China; 3 Digital Stomatology Research Institute, Peking University School of Stomatology, Beijing, China

**Keywords:** Computer-aided design, Computer-aided manufacturing, Removable space maintainer, Pediatric dentistry, Polyetheretherketone

## Abstract

**Background:**

The premature loss of primary teeth is a common problem in pediatric dentistry, resulting in disruption of the arch integrity. Hence, space maintainers (SM) used for maintaining spaces are necessary. However, current methods of making removable space maintainers (RSM) have some limitations.

**Methods:**

Digital models of dentition defects were obtained by using a scanning technique coupled with laser medical image reconstruction. The digital RSMs were designed using the 3Shape software. They were manufactured using two methods: polyetheretherketone (PEEK), and conventional methods (20 RSMs per group). For qualitative evaluation, the Likert five-point scale was used by 10 experts to obtain a score for 40 RSMs. The spaces between the tissue surfaces of the RSMs and the models were replaced using silicone, and the maximum and mean distances, as well as the standard deviation, were measured. A three-dimensional variation analysis was used to measure these spaces. The student’s t-test and Satterthwaite t-test were used to compare the differences in the spaces for the various materials.

**Results:**

The PEEK RSMs were found to fit the models well. In the qualitative assessment, the mean experts’ scores for the PEEK and conventional groups were 1.80 ± 0.40 and 1.82 ± 0.40, and there was no significant difference between the two groups (*p =* 0.875). In the quantitative assessment, the mean spaces for the PEEK digital RSMs and the conventional RSMs were 44.32 ± 1.75 μm, and 137.36 ± 18.63 μm, respectively, and the differences were statistically significant (*p <* 0.001). In addition, there were significant differences in the maximum space and the standard deviation between the two groups.

**Conclusion:**

Digitally designed and integrated RSMs were found to be superior to those produced using the conventional method. 3D variation analysis results showed that the mean distances and standard deviations of the PEEK groups were significantly smaller than those of conventional group (*p <* 0.01). A PEEK-manufactured RSM produced using CAD/CAM would be extremely suitable for clinical applications.

## Background

The premature loss of primary teeth is a common problem in the pediatric dentistry, resulting in the disruption of the arch integrity and adversely affecting the proper alignment of permanent successors [1]. Hence, space maintainer (SM) [2] are used for maintaining the space [3]. The removable space maintainer (RSM) is a kind of SM, designed for use with contiguous primary molar teeth loss, and its application is recommended by the profession [4]. There are several advantages to use an RSM [5], including maintaining the proximal, distal, and mesial lengths of a space, while maintaining the vertical height, thus restoring the aesthetics of the teeth [6], preventing speech disorders [7], and eliminating habits such as unilateral chewing. However, conventional RSMs incur some drawbacks [8], particularly in terms of their design and manufacture. For example, the manufacture of RSMs is complicated, because it is technically very sensitive, requires experienced technicians, and the product outcomes exhibit large individual variations. In addition, as the manufacture of RSMs features the use of curved snap rings and self-curing resin, it is difficult to ensure the precision of the snap rings within the space maintainers. During the polyreaction of the self-curing resin, shrinkage occurs [9], adversely affecting the fit between the tissue surface of the maintainer and the mucosa between the snap ring and the abutment. As a result, pediatric patients tend to adapt poorly to space maintainers. Furthermore, owing to the scarcity of artificial deciduous teeth products on the market, artificial permanent teeth products are usually modified to emulate the functionality of deciduous teeth; however, they do not accurately simulate their morphology. Hence, a precise, convenient, rapid design and manufacturing method is needed to address these problems.

Computer-aided design and manufacturing (CAD/CAM) has been used since the 1980s [10, 11]. It is now widely used in dentistry, for oral medicine, particularly in the field of prosthodontics [12–14]. When the same material is used with both CAD/CAM and traditional restoration techniques, the former yields more stable and durable results [15]. Currently, digital design techniques and rapid molding are typically used to design removable partial denture (RPD) metal scaffolds [16] and to manufacture their resin models [17], or to directly manufacture the RPD metal scaffold [18–23]. The present study set out to address the digital method of manufacturing an RPD.

PEEK [24] is a special engineering plastic with high rigidity and toughness [25], good flame-retardant properties, high mechanical performance [26], and high resistance to temperature, corrosion [27], and radiation. In addition, it offers a high level of biocompatibility [28] and resistance to fatigue caused by alternating stresses [29]. It is used in fixed prosthodontics, such as crown bridge repair and healing abutments, and to fabricate the scaffold and snap rings of an RPD. In addition, Ierardo et al. [30] conducted a pilot study using PEEK to fabricate an RSM with a dental CAD/CAM system, finding PEEK to be highly suitable for the fabrication of space maintainers.

Therefore, this study set out to investigate the application of CAD/CAM design to the RSMs used in pediatric dentistry and to evaluate the suitability of the technique for clinical applications.

## Materials and methods

### Construction of digital model for dentition defects

**Fig. 1:**
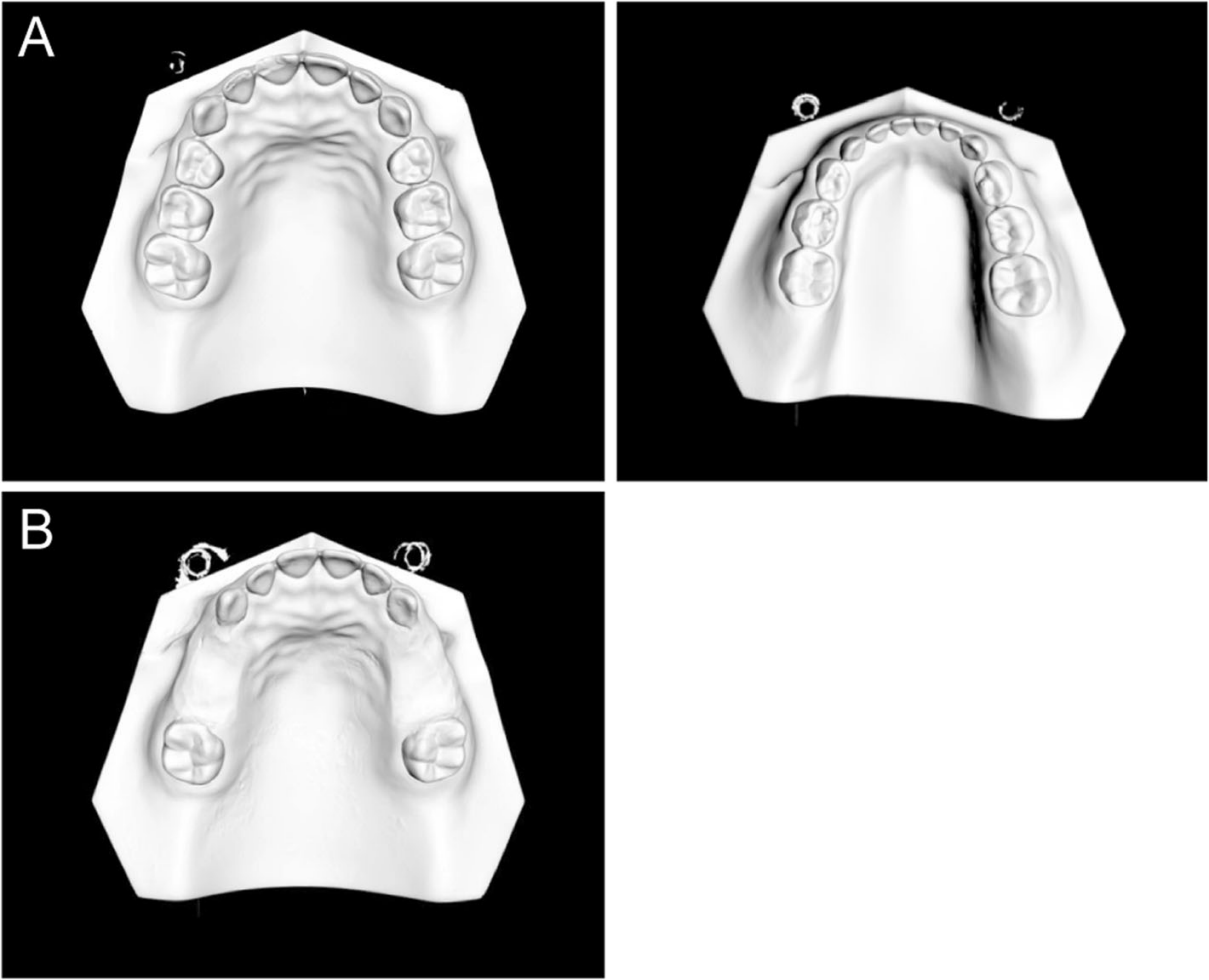
**a** Standard digital dental model; **b** standard digital defect dentition model for mandibular dentition

A standard negative model was used (Nissin™, China) to produce super-hard mixed dentition plaster (Type I dental plaster, Pegasus™, China) which was scanned using a three-dimensional (3D) model scanner (D800, 3Shape A/S, Denmark) to produce digital models (Fig. 1a). A partial-defect dentition model was constructed by removing the first and second deciduous molars from both sides of the standard model’s mandible. This standard model includes all the primary teeth and the four first molars. Then, a clear digital edentulism model was obtained by scanning the partial model (Fig. 1b). To ensure that the digital arrangement requirements for artificial teeth were satisfied by this model, the upper jaw model was also scanned and its occlusal relationship with the mandible model was evaluated.

### Digital design of RSMs

Dental CAD software (Dental System 2017, 3Shape A/S, Denmark) and reverse engineering software (Geomagic Studio 2014, Geomagic Inc., USA) were jointly used to design all the components of the RSM.

**Fig. 2:**
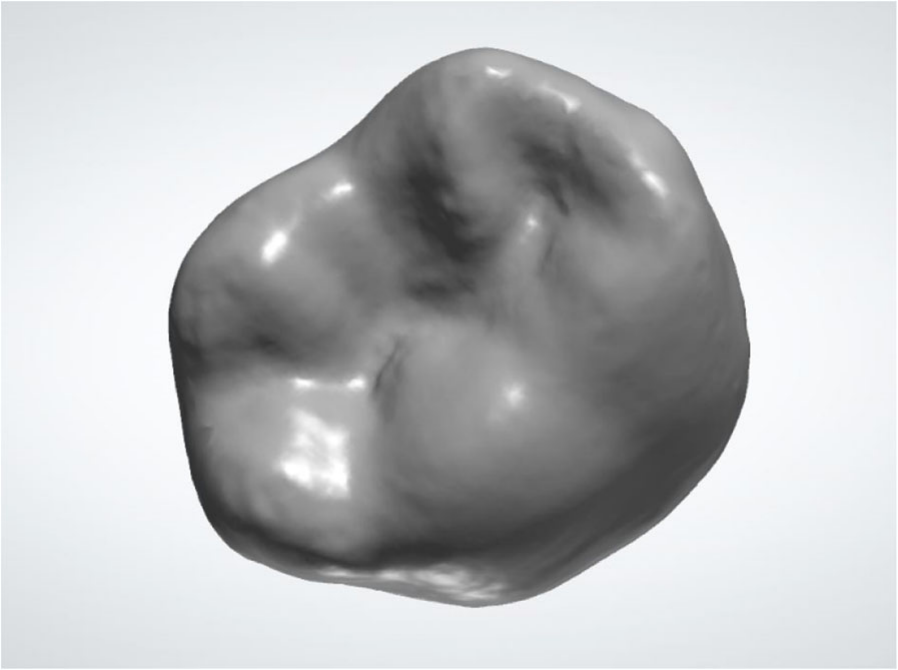
Artificial digitized model of the standard teeth crown of the mandibular right second molar tooth

As the 3Shape software database does not include deciduous teeth and given that the anatomical differences between deciduous and permanent molars are considerable, artificial digitized models of standard teeth crowns were constructed for this experiment. The complete standard digital jaw model data were then imported into the Geomagic Studio 2014 software and the “constructing sample boundary” function was used to define the boundary of each tooth. The processed data were then exported in stereolithography (STL) format. The anatomical and implant database in the anatomy element was selected from the control panel of the 3Shape software, and the new database was created by clicking “add” under the database menu and named “deciduous teeth database.” The model in the scan database was selected and the constructed teeth data were then imported into the database. The “Scan It Library” function was then used for tooth editing (Fig. 2). Bottom cutting, dental crown changing, dental crown shrinking, debris removal, and boundary and deformation point addition were all performed. Then, the models in the teeth database were edited. The teeth sequence and positions were arranged according to professional requirements and “smile editing” was performed. Hence, artificial digitized models of standard teeth crowns were constructed.

The RPD+ temporary tooth design module in 3Shape was selected. As RSMs are transitional prosthodontics, the selection of materials for RSMs should be different from that of RPD for adults. This is because during the growth and development phase, snap rings at the buccal side of the teeth are used minimally to avoid affecting the development of the width of the dental arch. Therefore, the effects of the maintainer base on the jaw and dental arch should be considered in the design, and the base should either be very short or have no vestibular base, and should instead have a long lingual base. Under conditions in which the mandible is fastened, snap rings would not be required, and fixation would be solely dependent on the lingual base.

**Fig. 3:**
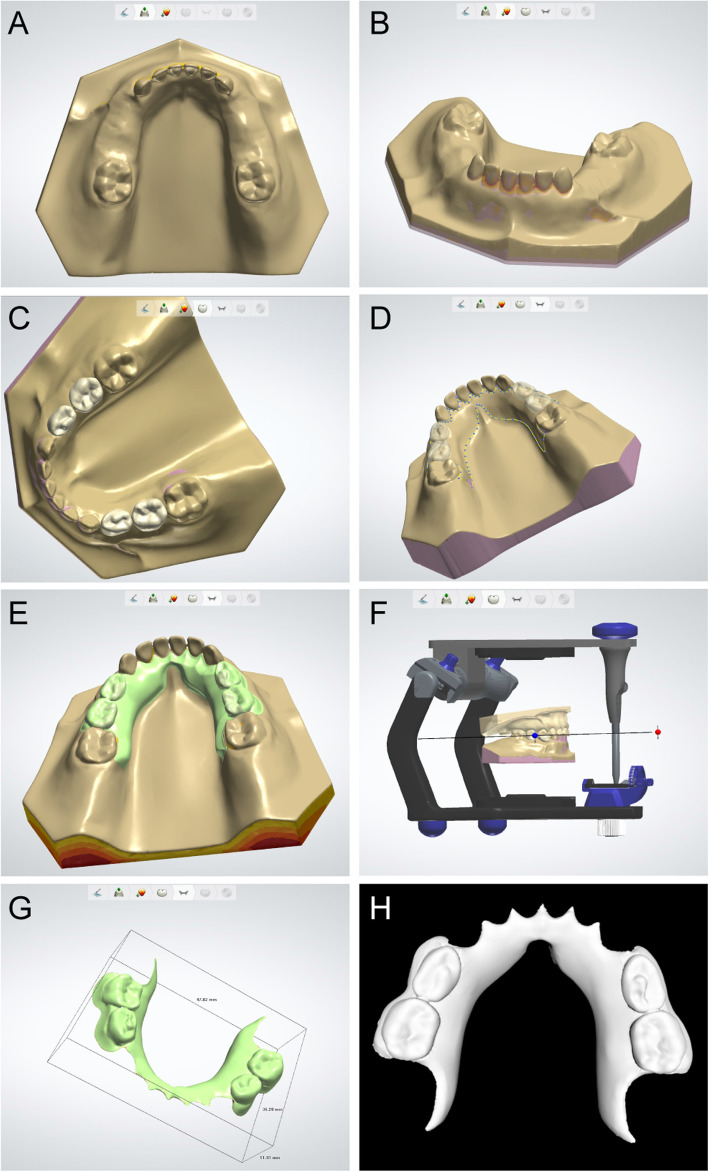
Digital design process for removable space maintainers; **a** importing the model data; **b** observing the model and filling in the voids [21]; **c** importing the artificial teeth model from the DIY deciduous teeth database into the software; **d** constructing the profile of the major connector; **e** composing the integrated removable space maintainer; **f** using the fictitious articulator to perform occlusal adjustment; **g** the final removable space maintainer; and (H) the final removable space maintainer data exported in STL format

The sites of missing teeth were labelled on the mandibular edentulism digital model, after which the digitized models of standard teeth crown were imported into the software. The “virtual articulator” function was used to adjust the anatomical module of the artificial teeth to ensure the functionality of the occlusion. The “major connector” design was chosen for the maintainer base and its thickness was set to the conventional value of 1.5 mm. Since the connector to the partial denture is relatively thin and represents the location where stress is most concentrated, its thickness was increased to 2.5 mm. The lingual side of the base was extended to the distal and middle parts of the first molar to form a base for the RSM. Finally, the finished set of RSM data were exported in STL format (Fig. 3a–h).

**Fig. 4:**
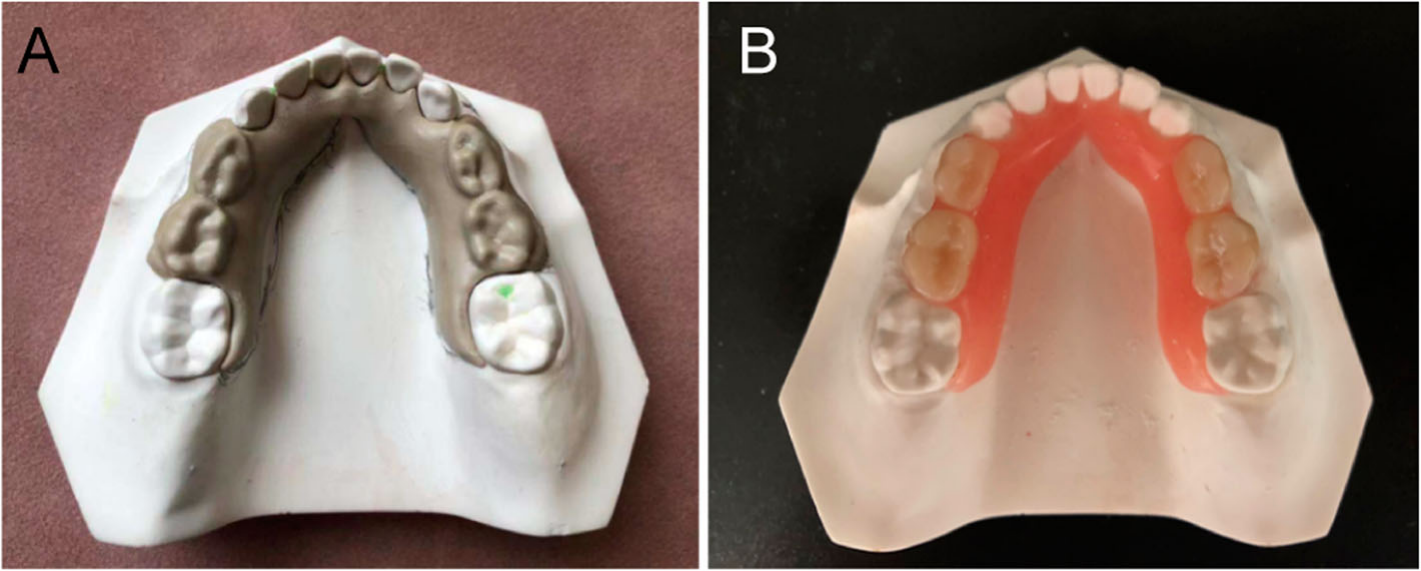
**a** Polyetheretherketone removable space maintainer; **b** laboratory fabrication of removable space maintainers

A 5-axis numerical-control milling machine (Organical Multi, R + K GmbH, Germany) was used to shape 20 PEEK material samples (Fig. 4a) into RSMs. After removing the support structure, the maintainer was polished and fitted onto a super-hard plaster model. Accordingly, a total of 40 super-hard plasters were fabricated. These were divided into two groups of 20.

The mandibular edentulism model described in Section 2.1 was used to fabricate the conventional RSM. The base was fabricated using Type 2 Class I denture base polymer powder (self-curing denture powder, 1R, biomimetic color, NISSIN™, Japan) and Heraeus three-layer synthetic resin teeth (Heraeus GmbH, Germany). After the maintainer was fabricated, it was fitted to a different super-hard plaster model (Fig. 4b).

### Suitability of different morphological components of RSMs

To qualitatively assess each RSM, the RSM was fitted to the mandibular plaster model, and then a preliminary evaluation of the model’s feasibility was performed through observations using a compression method. The assessment criteria for RPDs were used as a reference for the observations, based on the following principles proposed by Frank et al. [31], confirming that: [1] all occlusal rests were completely in place [2]; the rigid components of the denture contacted the corresponding abutment; and [3] no visible space greater than 1 mm existed between the main connector and the model. In this experiment, since no occlusal rests were involved, the second and third of Frank’s criteria were used for the assessment. In the compression method, a cement filler was used to compress the occlusal support vertically. The suitability was considered to be good if the denture did not significantly warp. Ten experts from the Department of Pediatrics of Peking University Hospital of Stomatology assessed the suitability of the 40 RSMs, assigning a score between 1 and 5 (Likert scale), where 1 was the most satisfactory and 5 was the least satisfactory.

For the quantitative assessment of RSMs, a silicone impression material (Variotime Light Flow, Heraeus GmbH, Germany) was injected onto the tissue surface of the RSM [32]. The RSM was then placed onto the super-hard plaster model, and a vertical force of 20 N was applied for 10 min until the silicone had completely solidified. Excess silicone was eliminated to prevent the removal of the RSM, thereby affecting the analysis results. The RSM was then removed, allowing the silicone gel to remain on the super-hard plaster model.

**Fig. 5:**
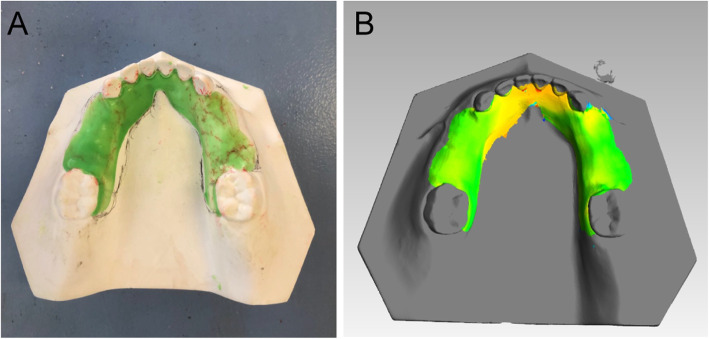
Quantitative assessment of removable space maintainers; **a** silicone film model data and (**b**) digital silicone film model data

The space between the denture and the model was then measured using digital analysis [33], and a dental scanner (Smart Optics, 880 GmbH, Germany) was used to obtain the corresponding digital silicone film model data. Sets of film model and raw digital model data were imported to the Geomagic software, and the data from the raw digital model were fixed as a reference. The digital silicone film model was set as the measurement subject. Three identical position points within the two models were selected for registration. The silicone film region in the digital silicone film model was selected as the measurement area and the “3D variation analysis” function was used to calculate the mean differences in the selected area (i.e., the differences in the mean thickness of the silicone film) between the two models. At the same time, a variation chromatogram was generated to display the thickness of the silicone film in various regions (Fig. 5 a, b). This analysis function was able to simultaneously calculate the maximum distance (i.e., the maximum thickness of the silicone film) for the two digital models; and these data were recorded as the “maximum space,” which was then used to express the maximum space between the denture tissue surface and the model. The “standard deviation” was recorded to express the uniformity of the space between the denture tissue surface and the model. This method was repeated for each of the forty model samples.

### Statistical analyses

Data management and statistical analysis were performed using the SPSS software program (version 22.0 IBM). Shapiro-Wilk and Bartlett tests were applied to the continuous variables. A student’s t-test was applied to the continuous variables to compare the PEEK and conventional groups for which the continuous variables satisfy the normal distribution and homogeneity of variance. Cohen’s d value was calculated for these effect sizes. A Satterthwaite t-test was applied to those continuous variables which were consistent with the normal distribution but not the homogeneity of variance. Glass’s △ value was calculated for these effect sizes. For the qualitative evaluation, 10 experts used the Likert five-point scale to apply a score to 40 RSMs. Kendall’s coefficient of concordance was used to determine the consistency of scoring. The average score assigned by the experts was used as the comprehensive expert score for the models. The power of the test was achieved using Proc power in the University Edition of the Statistical Analysis System (version SAS Studio 9.4). For the qualitative assessment of the RSMs, the null hypothesis was that there is no significant difference between the scores assigned by the experts to the two groups. For the quantitative assessment of the RSMs, the null hypothesis was that there is no significant difference in the mean distances, maximum distances, and standard deviations of the two groups. All *p* values were two-tailed, with a significance level of 0.05.

## Results

### Qualitative assessment results

The two types of RSMs exhibited a good fit onto the super-hard plaster standard model—there was tight contact between the denture base and the model, and between the major connecter and its lower plaster model; no 1 mm-spaces were observed and no significant warping was present. The compression of the RSM components using the cement filler also produced no significant warping, thus making them suitable for clinical applications.

The data is normally distributed (the results of the normality are listed in attached Table 1). Table 1 shows that the consistency evaluation of the experts for the PEEK group (Kendall = 0.556) is higher than that for the conventional group (Kendall = 0.484), which means that the suitability of the RMSs in the PEEK group were easier to assess and the scores were easier to converge.

**Table 1:** Comparison of consistency of experts’ evaluations of the two processing methods for the PEEK and conventional groups

Group	Kendall W	χ^2^	*p* value
PEEK	0.556	105.705	< 0.001
Conventional	0.484	92.037	< 0.001
All	0.498	194.263	< 0.001

**Fig. 6:**
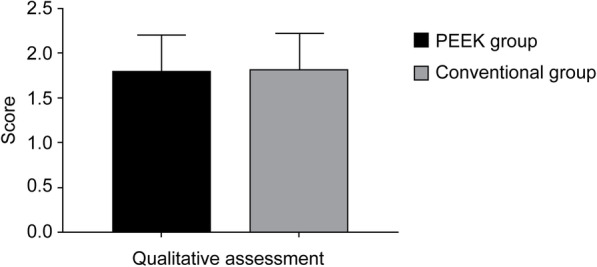
Quantitative assessment of removable space maintainers: there was no significant difference in the mean scores of the two groups

As listed in Table 2, the mean scores assigned by the experts for the PEEK and conventional groups were 1.80 ± 0.40 and 1.82 ± 0.40, respectively, while there was no significant difference between the two groups (*p =* 0.875). (Fig. 6).

**Table 2:** Comparison of experts’ comprehensive scores for the two processing methods for the PEEK and conventional groups

	Comprehensive experts’ score
PEEK group (*n =* 20)	1.80 ± 0.40
Conventional group (*n =* 20)	1.82 ± 0.40
Power	0.053
t value	−0.158
*p* value	0.875
Cohen’s d	0.050

### Quantitative assessment results

The data is normally distributed (the results of the normality test are listed in attached Table 2). Table 3 shows that the 3D variation analysis results revealed that the mean spaces for the PEEK digital RSMs and the conventional RSMs were 44.32 ± 1.75 μm, and 137.36 ± 18.63 μm, respectively, with the differences being statistically significant (*p <* 0.001). The maximum space values for the PEEK digital RSMs and the conventional RSMs were 602.71 ± 11.94 μm and 1261.88 ± 249.34 μm, respectively, while the differences were also statistically significant (*p <* 0.001). The standard deviation values for the PEEK digital RSMs and the conventional RSMs were 72.21 ± 5.72 μm and 361.22 ± 91.30 μm, respectively, while the differences were again statistically significant (*p <* 0.001).

**Table 3:** Comparison of markers for the two processing methods for the PEEK and conventional groups

	PEEK group	Conventional group	Power	t value	*p* value	Glass’s△ (95%CI)
Maximum distance	602.71 ± 11.94	1261.88 ± 249.34	> 0.999	11.809	< 0.001	2.64 (2.19–3.10)
Mean distance	44.32 ± 1.75	137.36 ± 18.63	> 0.999	22.241	< 0.001	4.99 (4.52–5.46)
Standard deviation	72.21 ± 5.72	361.22 ± 91.30	> 0.999	14.129	< 0.001	3.17 (2.70–3.63)

As listed in Table 3, for the maximum distances, mean distances, and standard deviation, the Glass’s △ were 2.64(2.19–3.10), 4.99(4.52–5.46), and 3.17(2.70–3.63), respectively. According to the relative and effective sizes of the differences shown in Table 4, the maximum distances, mean distances, and standard deviation of the two groups were large (effect size > 1.4). The power analysis is illustrated in attached Fig. 1.

**Table 4:** Effect size index

Relative Size	Effect Size	% of control group below the mean of the experimental group
	0.0	50%
Small	0.2	58%
Medium	0.5	69%
Large	0.8	79%
	1.4	92%

**Fig. 7:**
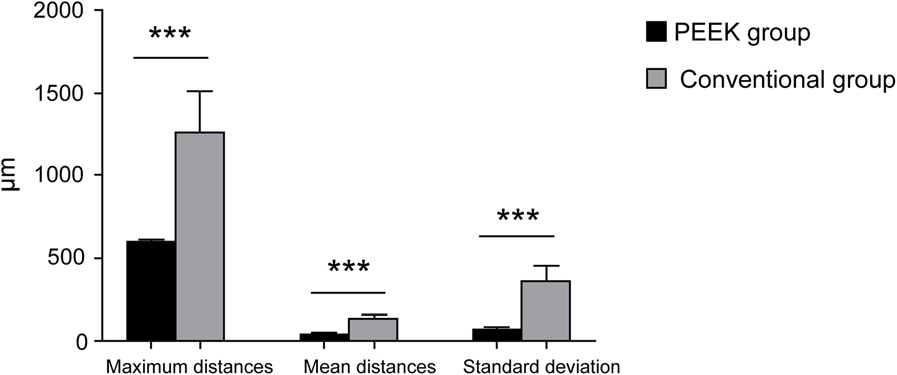
Differences in maximum distances, mean distances, and standard deviations between PEEK and conventional groups (*** indicates a significant difference)

As shown in Fig. 7, the maximum distances, mean distances and standard deviation of the PEEK group were significantly smaller than those of the conventional group, with the differences being statistically significant (*p <* 0.05).

## Discussion

An RSM offers multiple advantages; however, to date, it has presented problems in terms of its manufacture. Given that the denture base is composed of self-curing resin, shrinkage will occur as part of the curing process. The frictional resistance between the resin and the gypsum model partially inhibits the shrinkage. Thus, when the RSM cools to room temperature, latent stress will exist inside the base. Over the long term, this stress will be released slowly, resulting in the deformation of the base, the formation of micro cracks or cracks in and on the surface of the base resin, and even eventually lead to the fracture of the denture [34]. In this study, a new CAD/CAM method was used to help overcome these problems.

Currently, the application of CAD/CAM techniques to RPD manufacture focuses primarily on the design and manufacture of the scaffolds. Meanwhile, other denture components are still manufactured using the conventional processes. There are several related studies, such as that by Pooya et al. [35], who evaluated the overall accuracy and fit of conventional versus CAD/CAM RPD frameworks based on the results of a STL data analysis, and further evaluated the accuracy and fit of each component of the RPD framework. In a case report, Virard et al. [36] proposed an innovative procedure for producing an immediate removable denture, based on the use of three components: an intra-oral scanner, CAD with two different software used sequentially, and CAM with a 5-axis machine. The prosthodontics department at the Peking University Hospital of Stomatology [37] has reported on the case of a removable partial denture that was completely manufactured using CAD/CAM. Digital design has also been used in orthodontic dentistry, such as orthodontic space maintainers [38]. In this study, an update of the CAD/CAM software allowed us to use a new version of the 3Shape software, such that the frameworks and artificial teeth could be designed in the same design module, thus realizing a fully integrated design.

To the best of the authors’ knowledge, few studies have reported on the use of CAD/CAM to design and manufacture RSMs in pediatric dentistry. The use of this method could overcome the problems associated with traditional manufacturing. Such problems include the cost and the poor cooperation of children, as well as the size of intraoral scanners, in that they are too big to be applied to primary dentitions. In addition, there have been no reports detailing the use of CAD/CAM to design and manufacture RSMs. Studies into the area of digital technology being applied to space maintainers have also been rarely reported. Ji et al. [39] designed SM “band with loop” devices using CAD software, and these were manufactured by milling of PEEK blocks. Ierardo et al. (2017) fabricated orthodontic SMs in PEEK polymer through a digital workflow. However, they did not design or use artificial teeth. Soni [38] used digital methods to make a BruxZir zirconia SM for a six-year-old female patient with a chronic intra-radicular abscess in the upper right first primary molar which was treated by extraction. In this study, the technical route of using CAD/CAM to design and manufacture RSMs was explored, and the digitally prepared RSMs fitted the model well, which provided an important basis for clinical application.

In this study, PEEK was used to manufacture digital RSMs. This material has been widely used for the fabrication of clinical denture bases due to its good physiochemical, mechanical, and biological performance, and given that it may be easily cut and molded through the application of CAD/CAM technology. For children, it is safer and easier to make, and possibly superior to conventional methods which use artificial teeth and self-curing resin.

The present study found that, when using a CAD/CAM system, the maximum and mean distances and standard deviation for the PEEK group were significantly smaller than those for the conventional group, which indicates that the use of PEEK is promising for clinical applications. To qualitatively assess the compatibility of digitally designed RSMs, visual observation and a compression technique commonly used in clinical treatment and research were employed, and the results showed a good fit for the two types of RSMs. For quantitative assessment, reports by Stern et al. [40] and Dunham et al. [41] were referenced. They measured the space between the denture and the model to assess the former’s compatibility. According to the mean scores assigned by the experts, there was no significant difference between the two groups. This result may because the differences in the spaces were difficult to distinguish with the naked eye. Hence, to measure the space between the denture and the model, a 3D variation analysis commonly used to assess the suitability of fixed prostheses was employed [35]. The results showed that the maximum and mean distances and standard deviation for the PEEK group were significantly smaller than those for the conventional group, and these differences were statistically significant (*p <* 0.05). Therefore, the compatibility of the digital RSMs fabricated in this study is better than that of traditional types, largely because the conventional fabrication of RSMs is overly complex in terms of model construction; difficulties include the polymerization shrinkage of the self-curing resin, and the grinding and polishing of the RSM, which are prone to errors. Ye et al. [42] found that the space under a cast metal large connecter was 131.1 ± 87.1 μm (7.5–353.0 μm), while that under a PEEK large connecter was 52.8 ± 44.6 μm (0.5–177.4 μm). In this study, the mean space of the PEEK and conventional RMSs were 44.32 ± 1.75 μm and 137.36 ± 18.63 μm, respectively, and their values were all smaller than the above research results. The differences between the results of this study and the above research results may be caused by the progress of fabrication technology, the development of the associated equipment, the different structures of the RPD and RSM, and the different measurement areas.

In this study, digital design and fabrication simplified the manufacturing process, reduced deformation and errors, and improved the suitability of RSMs. Apart from the abovementioned three case reports, there have been no further clinical studies about the utilization of PEEK polymers in pediatric dentistry.

Currently, the fabrication cost of PEEK maintainers is high, and the color options are limited. When used in the frontal teeth region, the color of the base and the artificial teeth may affect the aesthetics. The color of PEEK maintainers can be improved by changing the performance of the material or by dyeing it. Future studies will focus on improving the color of the PEEK maintainers, the mechanical performance of various other RSMs, and seek out additional materials similar to PEEK. Moreover, research into the wear resistance of PEEK artificial teeth is also underway.

## Conclusion

In conclusion, the feasibility of CAD/CAM was examined to develop new PEEK-integrated RSMs and validated CAD/CAM, which provides a foundation for future clinical applications. This research has produced an original and creative method for combining digitalization technology and the clinical requirements of pediatric dentistry, thus opening up many new possibilities. The results of 3D variation analysis showed that the mean spaces for the PEEK digital RSMs and the conventional RSMs were 44.32 ± 1.75 μm, and 137.36 ± 18.63 μm, respectively, and the differences were statistically significant (*p <* 0.001). Design improvements and the search for new manufacturing materials require further research.

## Supplementary information

**Additional file 1 Attached** Fig. 1**.** (A) Power analysis for maximum distance. (B) Power analysis for mean difference. (C) Power analysis for standard deviation between PEEK (Group 1) and conventional (Group 2) groups.

**Additional file 2 Attached** Table 1**.** Normality test of data in qualitative assessment. **Attached** Table 2**.** Normality test of data in quantitative assessment

## Data Availability

The datasets acquired during and/or analyzed during the current study are available from the corresponding author on reasonable request.
