# Supplementary figures and images for: Computer-aided design of polyetheretherketone for application to removable pediatric space maintainers
Source: BMC Oral Health. 2020 Jul 10;20:201. doi: 10.1186/s12903-020-01184-6 (PMC7353737; doi:10.1186/s12903-020-01184-6)

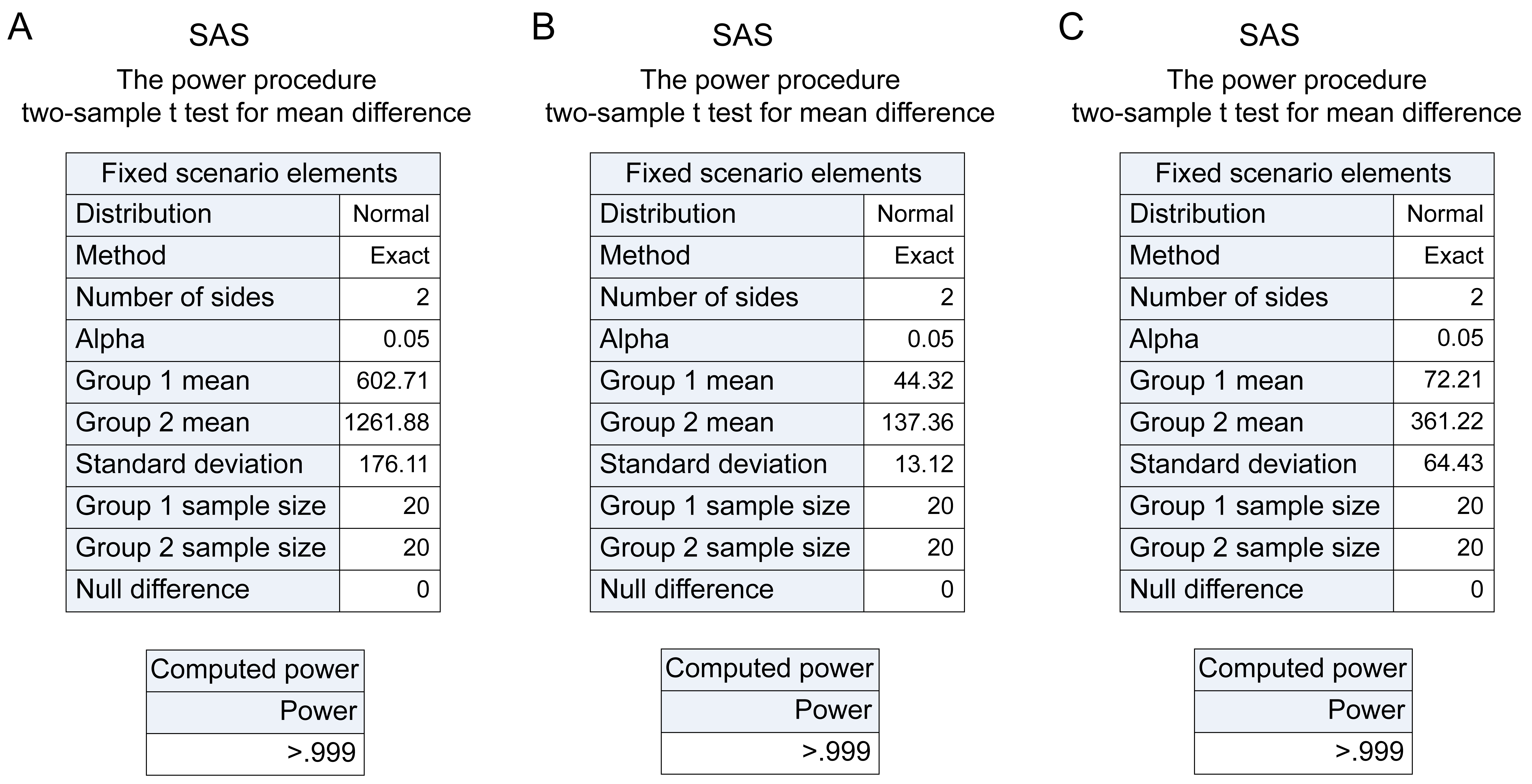

Supplement: Supplementary file 1 — Additional file 1 Attached Fig. 1. (A) Power analysis for maximum distance. (B) Power analysis for mean difference. (C) Power analysis for standard deviation between PEEK (Group 1) and conventional (Group 2) groups. [file 12903_2020_1184_MOESM1_ESM.tif]
