# Supplementary material for: Computer-aided design of polyetheretherketone for application to removable pediatric space maintainers
Source: BMC Oral Health. 2020 Jul 10;20:201. doi: 10.1186/s12903-020-01184-6 (PMC7353737; doi:10.1186/s12903-020-01184-6)
Supplement: Supplementary file 2 — Additional file 2 Attached Table 1. Normality test of data in qualitative assessment. Attached Table 2. Normality test of data in quantitative assessment [file 12903_2020_1184_MOESM2_ESM.docx]

**Attached Tables**

**Attached Table 1.** Normality test of data in qualitative assessment

|  | Shapiro-Wilk | | |
| --- | --- | --- | --- |
|  | Statistical magnitude | df | p value |
| PEEK group | 0.907 | 20 | 0.055 |
| Conventional group | 0.923 | 20 | 0.112 |

**Attached Table 2.** Normality test of data in quantitative assessment

| Group | | Shapiro-Wilk | | |
| --- | --- | --- | --- | --- |
|  |  | Statistical magnitude | df | p value |
| Maximum distance | PEEK | 0.907 | 20 | 0.055 |
|  | Conventional | 0.909 | 20 | 0.061 |
| Mean distance | PEEK | 0.960 | 20 | 0.546 |
|  | Conventional | 0.924 | 20 | 0.121 |
| Standard deviation | PEEK | 0.921 | 20 | 0.105 |
|  | Conventional | 0.931 | 20 | 0.161 |
| *. This is the lower limit of true significance. | | | | |
| a. Lilliefors significance correction. | | | | |
